# Rickettsial pathogens and their arthropod vectors.

**DOI:** 10.3201/eid0402.980205

**Published:** 1998

**Authors:** A F Azad, C B Beard

**Affiliations:** University of Maryland School of Medicine, Baltimore, USA. aazad@umaryland.edu

## Abstract

Rickettsial diseases, important causes of illness and death worldwide, exist primarily in endemic and enzootic foci that occasionally give rise to sporadic or seasonal outbreaks. Rickettsial pathogens are highly specialized for obligate intracellular survival in both the vertebrate host and the invertebrate vector. While studies often focus primarily on the vertebrate host, the arthropod vector is often more important in the natural maintenance of the pathogen. Consequently, coevolution of rickettsiae with arthropods is responsible for many features of the host-pathogen relationship that are unique among arthropod-borne diseases, including efficient pathogen replication, long-term maintenance of infection, and transstadial and transovarial transmission. This article examines the common features of the host-pathogen relationship and of the arthropod vectors of the typhus and spotted fever group rickettsiae.


### Rickettsial Pathogens and their Arthropod Vectors

**Abdu F. Azad* and Charles B. Beard†**
*University of Maryland School of Medicine, Baltimore, Maryland, USA; and
†Centers for Disease Control and Prevention, Atlanta, Georgia, USA

Rickettsial diseases, important causes of illness and death worldwide, exist primarily in endemic and enzootic foci that occasionally give rise to sporadic or seasonal outbreaks. Rickettsial pathogens are highly specialized for obligate intracellular survival in both the vertebrate host and the invertebrate vector. While studies often focus primarily on the vertebrate host, the arthropod vector is often more important in the natural maintenance of the pathogen. Consequently, coevolution of rickettsiae with arthropods is responsible for many features of the host-pathogen relationship that are unique among arthropod-borne diseases, including efficient pathogen replication, long-term maintenance of infection, and transstadial and transovarial transmission. This article examines the common features of the host-pathogen relationship and of the arthropod vectors of the typhus and spotted fever group rickettsiae.

Rickettsial diseases, widely distributed throughout the world in endemic foci with sporadic and often seasonal outbreaks, from time to time have reemerged in epidemic form in human populations. Throughout history, epidemics of louse-borne typhus have caused more deaths than all the wars combined (1). The ongoing outbreak of this disease in refugee camps in Burundi involving more than 30,000 human cases is a reminder that rickettsial diseases can reemerge in epidemic form as a result of the catastrophic breakdown of social conditions (2). In addition to explosive epidemics, sporadic but limited outbreaks of louse-borne typhus and other rickettsial diseases have been reported throughout the world. In the United States, a drastic increase of murine typhus in the 1940s, Rocky Mountain spotted fever (RMSF) in the late 1970s, and the human ehrlichioses in the 1990s attests to the potential emergence of these infections in populations at risk (3).

The rickettsiae's obligate intracellular existence in both mammalian and arthropod hosts serves as an excellent model for the study of complex host-parasite interactions. Rickettsial associations with obligate blood-sucking arthropods represent the highly adapted end-product of eons of biologic evolution. The ecologic separation and reduced selective pressure due to these associations may explain rickettsial genetic conservation. Their intimate relationships with vector hosts (Table 1) are characterized by efficient multiplication, long-term maintenance, transstadial and transovarial transmission, and extensive geographic and ecologic distribution. Although rickettsiae have a symbiotic relationship with their arthropod hosts, in some instances, they act as true parasites—for example, members of the *Wolbachia* and *Orientia tsutsugamushi* alter reproduction and manipulate cellular processes in their arthropod hosts (4), and the agent of epidemic typhus, *Rickettsia prowazekii*, kills its vector, the human body louse (5).

Although rickettsiae are maintained in nature through arthropod vectors, they frequently infect vertebrates, which in turn allow new lines of vectors to acquire infection from the rickettsemic hosts. The involvement of vertebrates, including humans, in the rickettsial cycle is variable and in some cases complicated (Figure). With the exception of epidemic typhus, humans are not essential in the rickettsial cycle. Humans acquire rickettsial infection from the infected vectors. While tick-borne rickettsiae are transmitted to humans by tick salivary secretions, flea- and

Address for correspondence: Abdu F. Azad, Department of Microbiology and Immunology, University of Maryland School of Medicine, 655 W. Baltimore Street, Baltimore, MD 21201 USA; fax: 410-706-0282; e-mail: aazad@umaryland.edu.

Table 1. Epidemiologic features of the pathogenic rickettsiae[a]

| *Rickettsia* species | Disease | Natural cycle[b] Vectors | Hosts | Geographic distribution |
|---|---|---|---|---|
| Typhus group: | | | | |
| *Rickettsia prowazekii* | Epidemic typhus | Human body lice | Humans | Worldwide |
| | Recrudescent typhus | None | Humans | Worldwide |
| | | Lice, fleas | Flying squirrels | Eastern USA |
| *R. typhi* | Murine typhus | Fleas | Rodents | Worldwide |
| | | Fleas | Opossums | USA |
| *R. felis* | Murine typhuslike | Fleas | Opossums | USA |
| Spotted Fever group: | | | | |
| *R. rickettsii* | Rocky Mountain spotted fever | Ticks | Small mammals, dogs, rabbits, birds | North & South America |
| *R. conorii* | Boutonneuse fever | Ticks | Rodents, dogs | Africa, Southern Europe, India |
| *R. sibirica* | North Asia tick typhus | Ticks | Rodents | Eurasia, Asia |
| *R. japonica* | Japanese spotted fever | Ticks | Rodents, dogs | Japan |
| *R. australis* | Queensland tick typhus | Ticks | Rodents | Australia |
| *R. akari* | Rickettsialpox | Mites | House mice, rats | Worldwide |
| Ehrlichioses group: | | | | |
| *Ehrlichia chaffeensis* | Human monocytic ehrlichiosis | Ticks | Humans, deer | USA, Europe |
| *Ehrlichia* Sp | Human granulocytic ehrlichiosis | Ticks | Humans, deer, rodents | USA, Europe |
| Others: | | | | |
| *Coxiella burnetii* | Q fever | Ticks | Small mammals, sheep, goats, cattle, dogs | Worldwide |
| *Orientia tsutsugamushi* | Scrub typhus | Mites | Rodents | Asia, Indian subcontinent, Australia |

[a]Not listed are *R. helvetica, R. honei,* and *R. slovaca* (6).
[b]Evidence for arthropod serving as a vector or vertebrate serving as a host is based on the rickettsial isolation from field-collected specimens, experimental studies, and indirect evidence for rickettsial presence or exposure to rickettsiae (hemolymph test and serosurveys).

louse-borne rickettsiae are transmitted to humans through contamination of broken skin and mucosal surfaces by infected vector feces.

Although rickettsiae have common features with their vertebrate and invertebrate hosts, they differ considerably in terms of arthropod vectors, geographic distribution, and virulence (Table 1). In this article, we focus on the members of the typhus group (TG) and spotted fever group (SFG) rickettsiae to construct a conceptual framework of the natural history of human rickettsioses and evaluate feeding behavior of the vectors with regard to rickettsial maintenance and transmission.

### Tick-borne Rickettsial Pathogens

The development and extensive use of the hemolymph test (which has been fundamental to tick and rickettsial surveys), improved isolation methods, and the application of molecular techniques have helped identify 14 valid, relatively distinct SFG rickettsiae (Tables 1, 2). Except for mite-borne *R. akari*, all SFG rickettsiae are transmitted by ixodid ticks (Tables 1, 2). In addition to *R. rickettsii*, the etiologic agent of RMSF, eight other tick-borne rickettsial species are human pathogens (Table 1; 6). The remaining SFG rickettsiae are isolated only from ticks and have low or no pathogenicity to humans or certain laboratory animals (7). However, some of these rickettsiae could be etiologic agents of as-yet-undiscovered, less severe rickettsioses.

Distribution of SFG rickettsiae is limited to that of their tick vectors. In the United States, a high prevalence of SFG species in ticks cannot be explained without the extensive contributions of transovarial transmission. The transovarial and transstadial passage of SFG rickettsiae within tick vectors in nature ensures rickettsial survival without requiring the complexity inherent in an obligate multihost reservoir system. Although many genera and species of ixodid ticks are naturally infected with rickettsiae, *Dermacentor andersoni* and *D. variabilis* are the major vectors of *R. rickettsii*. SFG infection rates vary considerably by state. For example, the infection rate for adult *D. variabilis* collected from vegetation and mammalian hosts was 2% to 9% in Connecticut, 5% in New York, 6% in Kentucky-Tennessee and Maryland, 8.8% in Arkansas, and 10% in Alabama (5,8,9). Rickettsia and tick surveys indicate that *R. rickettsii* is less prevalent in vector ticks than some other SFG rickettsiae. In most cases, the SFG-positive ticks, including *D. andersoni* and *D. variabilis*, are infected with nonpathogenic rickettsiae rather than with *R. rickettsii* (Table 2). The low prevalence of *R. rickettsii* in SFG-positive ticks is intriguing. Interspecific competition among ticks may result in stable maintenance of SFG rickettsiae through transovarial transmission and may cause the gradual replacement of *R. rickettsii* in the tick population. Very little is known about the process of interspecific competition between prokaryotic microorganisms in ticks. Burgdorfer et al. (10) reported that *D. andersoni* from the east side of Bitterroot Valley in western Montana contained a nonpathogenic SFG-rickettsia, which they named East Side agent. East Side agent has recently been described as a new species, *R. peacockii* (11). This rickettsia is rarely present in tick hemolymph and is readily missed by the standard hemolymph test. The rickettsiae are confined primarily to the tick posterior diverticulae of the midgut, the small intestine, and most importantly, the ovaries. *R. peacockii* is maintained in the tick population through transovarial transmission, and the infected ticks were shown to be refractory to ovarian infection with *R. rickettsii*. However, these ticks acquired experimental infection with *R. rickettsii* and transmitted rickettsiae horizontally (10,11). Thus, ticks constitutively infected with *R. peacockii* and infected experimentally with *R. rickettsii* were unable to transmit *R. rickettsii* to their progeny. In effect, infection of *D. andersoni* with *R. peacockii* blocked the subsequent ability of the ticks to transmit *R. rickettsii* transovarially (10,11). The phenomenon of transovarial interference provided an explanation for the curious long-standing disease focus in Bitterroot Valley. Most cases of RMSF have occurred among residents on the west side of the valley where *D. andersoni* were abundant; on the east side, *D. andersoni* were also abundant and were reported

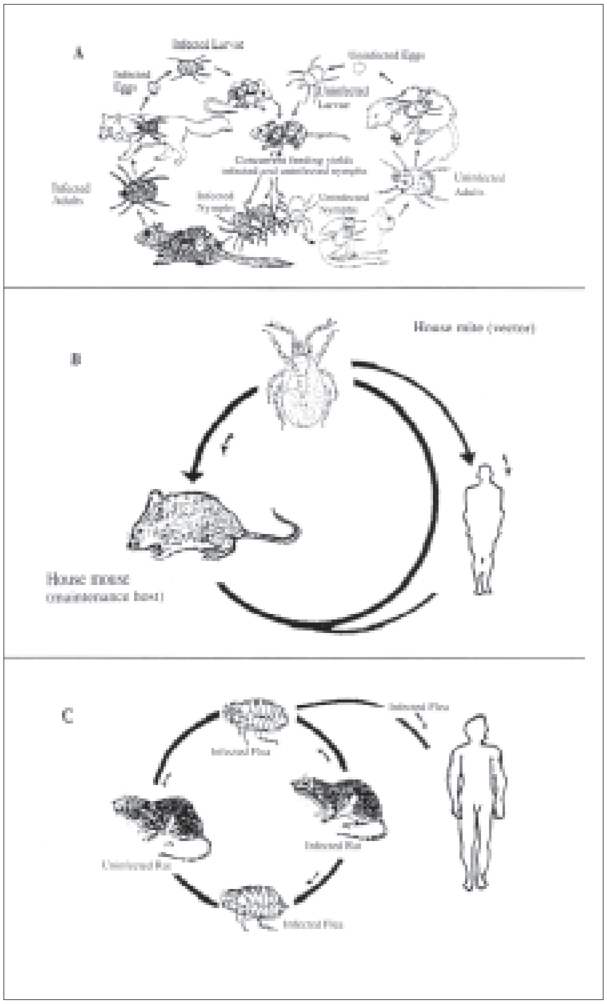

Figure. Composite diagram of the life cycle of Rocky Mountain spotted fever, rickettsialpox, and murine typhus. A. Life cycle of *Rickettsia rickettsii* in its tick and mammalian hosts (7); B. *Rickettsia akari* life cycle; and C. *Rickettsia typhi* life cycle.

Table 2. Epidemiologic characteristics of the North American tick-borne *rickettsia*[a]

| Rickettsia species | Disease | Natural cycle: Vectors | Natural cycle: Hosts |
|---|---|---|---|
| *Rickettsia rickettsii* | Rocky Mountain spotted fever | *Dermacentor, Amblyomma, Rhipicephalus, Haemaphysalis* | Small mammals, dogs, rabbits, birds |
| *R. akari* | Rickettsialpox | *Liponyssoides* | House mice, rats |
| *R. amblyommii* | | *A. americanum* | Small mammals |
| *R. bellii* | | *D. andersoni, D. variabilis, D. occidentalis, D. albopictus, H. leporispalustris* | Rodents, dogs |
| *R. canada* | | *Haemaphysalis* | Rabbits, hares, birds |
| *R. montana* | | *D. andersoni, D. variabilis* | Rodents, dogs |
| *R. parkeri* | | *A. americanum, A. maculatum* | Domestic animals, birds, rodents |
| *R. peacockii* | | *D. andersoni* | Rodents, deer |
| *R. rhipicephali* | | *R. sanguineus, D. andersoni, D. variabilis, D. occidentalis* | Small mammals |

[a]Excluding four as yet undescribed species of SFG rickettsiae (WB-8-2, 364-D, Tillamook, and the *D. parumapertus* agent).

to feed on residents, yet few (if any) locally acquired cases of RMSF occurred there (8,10). In the presence of *R. peacockii*, *R. rickettsii* could not be maintained transovarially—it could only be transmitted horizontally, and thus long-term maintenance could not be sustained. Transovarial interference by tick-associated symbionts such as *R. peacockii* is unlikely to be confined only to *D. andersoni* ticks on the east side of Bitterroot Valley, Montana. Burgdorfer et al. (10) stated that transovarial interference of *R. rickettsii* in *D. andersoni* ticks may be mediated by other nonpathogenic SFG rickettsiae—*R. montana* and *R. rhiphicephali*. Tick surveys for SFG rickettsiae generally report finding *R. rickettsii* only in a minority of ticks with rickettsiae (Table 3). Most infected ticks harbor nonpathogenic species. Thus, transovarial interference may have epidemiologic significance: it may explain why ticks collected from various geographic regions are not infected with two or more species of SFG rickettsiae.

The tick-rickettsia interrelationships are complex, and the mechanisms used by rickettsiae to survive in unfed overwintering ticks or during molting are poorly understood. How changes (e.g., before and after blood meal) in tick gut physiology influence rickettsial growth, cell division, and differential expression of rickettsial surface protein is not well understood. Although experiments (8,10) have deciphered the phenomenon of reactivation of rickettsial virulence after infected ticks take a blood meal, the underlying molecular events have yet to be elucidated. Other aspects of rickettsia-tick interactions need to be studied. For example, after feeding, a tick larva enters a quiescent period before emerging as a questing nymph the following year. How rickettsiae survive within the tick during this quiescent period and regain infectivity during the nymphal feeding is poorly understood. Although the precise mechanisms of rickettsial reactivation are not known, temperature shift and blood intake are believed to reactivate rickettsiae. As in the *Borrelia burgdorferi-Ixodes scapularis* model, transmission of rickettsiae probably cannot occur until after 24 hours of tick attachment, which allows time for rickettsial growth.

### Insect-Borne Rickettsial Pathogens

Unlike SFG, TG rickettsiae are associated with insects (Table 1). Their association with blood-sucking insects such as human body lice (*Pediculus humanus*) and fleas (*Xenopsylla cheopis* and other rodent fleas [13-15]) provides rickettsiae the potential to spread rapidly among susceptible populations. Both fleas and human body lice, intermittent feeders, are capable of multiple feeding and thus of transmitting rickettsiae to several hosts. Outbreaks of epidemic typhus thereby can result from rapid transmission of *R. prowazekii* from human to human by infected lice. Unlike ticks that transmit SFG rickettsiae, lice infected with *R. prowazekii* die within 2 weeks after imbibing infected blood. *R. typhi*, the etiologic agent of murine typhus, does not shorten the life span of fleas (15). Although *R. typhi* and *R. felis* are maintained transovarially in fleas (15-18), there is no evidence that *R. prowazekii* is maintained in human body lice vertically. Since lice die of *R.*

*prowazekii* infection, the role of the reservoir in maintaining the rickettsiae in nature becomes essential. *R. prowazekii* sequesters in its human host; persistence of rickettsiae occurs despite the strong, long-lasting immunity after infection with *R. prowazekii*. Patients with recrudescent typhus (Brill-Zinsser disease) serve as potential reservoirs capable of infecting lice. Although a search for a zoonotic cycle of *R. prowazekii* in areas with louse-borne typhus epidemics (e.g., Ethiopia and Burundi) proved to be unsuccessful, flying squirrels (*Glaucomys volans*) in the eastern United States are naturally infected with this organism. Flying squirrel ectoparasites (lice and fleas) were implicated in the transmission of *R. prowazekii* between the squirrels and from squirrels to humans (5). Although the distribution of *G. volans* extends to the entire eastern United States as well as to isolated areas of Mexico and Guatemala, the search for an extrareservoir of *R. prowazekii* was not pursued further. Consequently, the importance of the *R. prowazekii* and squirrel system remains unclear. In the absence of a zoonotic cycle, conditions such as widespread lice infestations, active human infection, reactivation of latent infection in patients with recrudescent typhus could easily ignite a resurgence of louse-borne typhus. Louse-borne typhus continues to occur in epidemics following the breakdown of social, economic, or political systems, as exemplified by recent outbreaks in Burundi and remote parts of South America. Therefore, active surveillance to monitor louse-borne typhus and prevent its spread is indicated.

In contrast to louse-borne typhus, murine typhus is prevalent throughout the world and accounts for widespread illness in areas infested with many rats and fleas. Murine typhus occurs in epidemics or has a high prevalence, is often unrecognized and substantially underreported, and although it may be clinically mild, can cause severe and even fatal cases (19). Thousands of human cases used to occur annually in the United States (13,14). Outbreaks have been reported in Australia and recently in China, Kuwait, and Thailand (13-15). The classic cycle of *R. typhi* involves rats (*Rattus rattus* and *R. norvegicus*) and primarily the rat flea, *X. cheopis* (13,14). *X. cheopis* is the main vector, and transmission is affected by contact with rickettsia-containing flea feces or tissue during or after blood feeding. Reported cases of murine typhus in the United States are from south and central Texas and the Los Angeles and Orange County area of California (21-25). However, most of the cases are attributed to opossum-cat flea cycles. Both opossums and domestic cats collected from the case areas were seropositive for *R. typhi* antibodies. The cat flea, *Ctencephalides felis*, which is a competent vector of murine typhus, is the most prevalent flea species (97%) collected from opossums, cats, and dogs in southern Texas; no fleas were recovered from rats in this area. In addition to *R. typhi*, *R. felis* was also found in opossums and their fleas (15). This finding, consistent with surveys in other areas of the country (14,20), further documents the reduced role of rat and *X. cheopis* in the maintenance of murine typhus within endemic-disease areas of the United States. The maintenance of *R. typhi* in the cat flea and opossum cycle is therefore of potential public health importance since *C. felis* is a prevalent and widespread pest that avidly bites humans (12,15).

Table 3. Species composition of tick-borne rickettsiae isolated from hemolymph-positive *Dermacentor* ticks[a]

| Rickettsial sp. | California *D. occidentalis* (No. isolates) | Montana *D. andersoni* (No. isolates) | Ohio *D. variabilis* (No. isolates) | Long Island *D. variabilis* (No. isolates) | Maryland *D. variabilis* (No. isolates) |
|---|---|---|---|---|---|
| *R. rickettsii* | 0 (0) | 9 (10) | 18 (4) | 0 (0) | 8 (2) |
| *R. rhipicephali* | 96 (79) | 44 (47) | 0 (0) | 0 (0) | 0 (0) |
| *R. montana* | - | 7 (8) | 59 (13) | 100 (100) | 0 (0) |
| Other SFG[b] | - | - | 5 (1)[c] | - | 88 (23)[d] |
| *R. bellii* | 4 (3) | 39 (41) | 18 (4) | 0 (0) | 4 (1) |
| Total number isolates | 82 | 106 | 22 | 100 | 26 |

[a]Shows a compilation of various statewide surveys, comparing the species composition of SFG rickettsiae in *Dermacentor spp.* ticks that tested positive by immunofluorescence assay.
[b]SFG, spotted fever group.
[c]*R. amblyommii.*
[d]Mouse anti-sera made against Maryland isolates reacted with WB-8-2 (unnamed SFG rickettsiae).

In many parts of the world, murine typhus infection is intimately associated with introduced commensal rodents (*R. rattus*, *R. norvegicus*, and *Mus musculus*) and their ectoparasites, particularly fleas. Although *R. typhi* have been isolated from other commensal rodents and even shrews, they do not seem to play a role in the transmission of murine typhus to humans (13,14). In Rangoon (Myanmar), of four species of murines and one shrew commonly found in buildings, 7% (*M. musculus*) to 30% (*R. rattus*) and 38% (*Bandicota bengalensis*) of those tested were seropositive to *R. typhi*. In contrast, 62% of *R. rattus* collected from buildings in Addis Ababa (Ethiopia) and 49% of those collected in Sarawak (Nepal) were seropositive for murine typhus (Azad et al., unpub. data). Infection rates in *X. cheopis* fleas collected from rats were 7% to 18%. While infection rates vary considerably among indoor rats and their fleas, murine typhus infection seems clearly associated with indoor rat populations throughout the world. However, in the absence of indoor rats, murine typhus infection is maintained in suburban and rural cycles when native animals seek shelter in human habitations where food and hospitable environments are plentiful.

### Pathogen-Arthropod Interaction

The process of displacement of pathogenic rickettsiae with nonpathogenic endosymbionts in ticks through transovarial interference is of potential epidemiologic importance. The displacement might occur only if transovarial maintenance of pathogenic rickettsiae harms the host or the maintenance of nonpathogenic organisms confers important advantages to the tick progeny. Burgdorfer et al. (10), in experimental studies with the *D. andersoni*-*R. rickettsii* model, observed that maintenance of this pathogen in ticks over several generations resulted in unusually high death rates among engorged females and reduced numbers and fertility of deposited eggs. If tick infection with pathogenic rickettsiae in nature adversely affected egg maturation, oviposition, and embryogenesis, the balance would favor a tick population infected with the nonpathogenic rickettsiae, and over time such tick populations would displace those infected with *R. rickettsii*. Also, ticks infected experimentally with *R. montana* and *R. rhipicephali* could not maintain *R. rickettsii* through transovarial transmission, which suggests an interference phenomenon. Such a precedent exists—several tick species carry nonpathogenic rickettsiae (e.g., *D. andersoni* in east side Bitterroot Valley of western Montana, *A. americanum* in Maryland) frequently encountered in tick samples from different parts of the United States (8,9).

Recent work in our laboratory with TG rickettsiae suggests that interspecific competition between closely related rickettsiae may control rickettsial establishment in arthropods. Studies have identified both *R. typhi* and *R. felis* in opossums and in their cat fleas in endemic-disease regions in Texas and California (21-25). However, infection with both rickettsiae has not been observed in individual fleas (20-24). The intermittent feeding behavior of cat fleas associates them with various hosts, which increases the likelihood of infection with more than one pathogenic organism. Cat fleas constitutively infected with *R. felis* and experimentally infected with *R. typhi* contained both rickettsial species. While the *R. felis* infection rate in the infected flea population was 86% to 94%, prevalence of dually infected fleas was 13% and 26% (25). While no other relationships involving multiple bacterial infections in arthropods have been studied as thoroughly, the results from the few available studies show that dual infections in arthropod vectors are rare (e.g., human granulocytic ehrlichia was recently identified in 2.2% and 4% of *I. scapularis* ticks coinfected with *B. burgdorferi*) (26).

Whether infection with nonpathogenic rickettsiae presents an advantage to the tick population is difficult to ascertain because of lack of experimental data. There is no experimental evidence for rickettsial-induced postzygotic reproductive incompatibility, parthenogenesis, and feminization of genetic males as observed for members of the genus Wolbachia (4,27). Although a rickettsial relative is associated with male killing in the ladybird beetle (27), we do not know whether any nonpathogenic members of the SFG rickettsiae can induce reproductive incompatibility or sex distortion in ticks. A compilation of data from various laboratories that maintain cat flea colonies indicate that after several generations *R. felis* infection in fleas approaches 100% (28). Whether the high infection rate is the result of selection through reproductive incompatibility remains to be elucidated. Flea samples from opossums, cats, and dogs in different parts of the

United States were infected with *R. felis*. Since it is maintained transovarially, *R. felis* could be used as a marker to follow changes in the infection rates over time.

Several questions remain: 1) why does infection with nonpathogenic rickettsiae prevent establishment of virulent species in the ovaries of infected ticks; 2) what are the molecular bases for transovarial interference; 3) are nonpathogenic rickettsiae selected favorably by their arthropod hosts through reproductive incompatibility; and 4) why are nonpathogenic rickettsiae found more often in ticks than a virulent species.

### Summary and Conclusions

Ixodid ticks, fleas, and lice are temporary obligate ectoparasites often found on the same vertebrate hosts. They differ substantially with respect to feeding behavior and digestion of blood meals (30), which can affect rickettsiae transmission from vector to vertebrate hosts. While all stages of ticks and lice are blood feeders, only adult fleas take blood from the host. Fleas and lice feed intermittently and digest blood meals rapidly, while ixodid ticks feed for several days and digest meals very slowly (29,30). SFG rickettsiae are associated with ixodid ticks, while TG rickettsiae are transmitted by fleas and lice. Efficient transovarial and transstadial transmission of rickettsiae in ticks ensures rickettsial survival while maintaining the genetic integrity of the SFG rickettsiae. This mechanism also allows ticks to serve as reservoir host for SFG rickettsiae. The SFG rickettsiae are transmitted by tick bites, whereas TG rickettsiae are deposited with insect feces at the bite site. At each life stage (larva, nymph, and adult), ticks feed only once; if undisturbed, they transmit rickettsiae to a single host, whereas repeated feedings allow fleas and lice to infect several hosts. Thus, vector feeding behavior accounts for the observed differences in disease epidemiology and natural history between tick- and insect-borne rickettsioses. Despite their strong similarities, SFG and TG rickettsiae have major differences in terms of growth, entry, and exit from host cells. Rickettsial characteristics related to vector compatibility were discussed in a recent publication by Hackstadt (31).

The molecular aspects of rickettsia-vector interactions are not well understood largely because only a few laboratories have addressed the mechanisms by which arthropods and rickettsiae interact. These intracellular organisms have long been associated with arthropods, and yet we know very little about their relationship with arthropod vectors.

This work was supported by grants R37 AI 17828 and R01 AI 43006 from the National Institutes of Health.

Dr. Abdu Azad is professor of microbiology and immunology, University of Maryland School of Medicine, Baltimore. His laboratory research focuses on host parasite interactions with particular interest in the molecular aspects of rickettsial pathogenesis.

Dr. Charles Ben Beard is research entomologist and chief, Vector Biology Activity, Entomology Branch, Division of Parasitic Diseases, NCID, CDC. Research in his laboratory focuses on the molecular biology of insect disease vectors and the molecular epidemiology of opportunistic infections in persons with AIDS.

### References

1. Zinsser H. Rats, lice and history. Boston: Little, Brown and Co.;1934.
2. Raoult D, Roux V, Ndihokubwayo JB, Bise G, Baudon D, Martet G, et al. Jail fever (epidemic typhus) outbreak in Burundi. Emerg Infect Dis 1997;3:357-60.
3. Walker DH, Barbour A, Oliver JH, Dumler JS, Dennis DT, Azad AF, et al. Emerging bacterial zoonotic and vector-borne diseases: prospects for the effects on the public health. JAMA 1996;275:463-9.
4. Werren JH. Biology of Wolbachia: Annu Rev Entomol 199742:587-609.
5. Azad AF. Relationship to vector biology and epidemiology of louse and flea-borne rickettsioses. In: Walker DH, editor. Biology of rickettsial diseases. Boca Raton (FL): CRC Press; 1988. p. 52-62.
6. La Scola B, Raoult D. Laboratory diagnosis of rickettsioses: current approaches to diagnosis of old and new rickettsial diseases. J Clin Microbiol 1997;35:2715-27.
7. McDade JE, Newhouse VF. Natural history of *Rickettsia rickettsii*. Ann Rev Microbiol 1986;40:287-309.
8. Burgdorfer W. Ecological and epidemiological consideration of Rocky Mountain spotted fever and scrub typhus. In: Walker DH, editor. Biology of Rickettsial Diseases. Boca Raton (FL): CRC Press; 1988. p. 33-50.
9. Schriefer ME, Azad AF. Ecology and natural history of *Rickettsia rickettsii*. In: Sonenshine DE, Mather TN, editors. Ecological dynamics of tick-borne zoonoses. Oxford: Oxford University Press;1994.
10. Burgdorfer W, Brinton PL. Mechanisms of transovarial infection of spotted fever rickettsiae in ticks. Ann New York Acad Sci 1975;266:61-72.
11. Niebylski ML, Schrumpf ME, Burgdorfer W, Fischer ER, Gage KL, Schwan TG. *Rickettsia peacockii* sp nov., a new species infecting wood ticks, *Dermacentor andersoni*, in western Montana. Int J Sys Bacteriol 1997;47:446-52.
12. Azad AF. Epidemiology of murine typhus. Annu Rev Entomol 1990;35:553-69.

13. Traub R, Wisseman CL Jr, Azad AF. The ecology of murine typhus: a critical review. Trop Dis Bull 1978;75:237-317.
14. Azad AF, Radulovic S, Higgins JA, Noden BH, Troyer MJ. Flea-borne rickettsioses: some ecological considerations. Emerg Infect Dis 1997;3:319-28.
15. Azad AF, Sacci JB Jr, Nelson WM, Dasch GA, Schmidtman ET, Carl M. Genetic characterization and transovarial transmission of a novel typhus-like Rickettsia found in cat fleas. Proc Natl Acad Sci U S A 1992;89:43-6.
16. Azad AF, Traub R, Baquar S. Transovarial transmission of murine typhus rickettsiae in *Xenopsylla cheopis*. Science 1985;227:543-5.
17. Adams WH, Emmons RW, Brooks JE. The changing ecology of murine (endemic) typhus in southern California. Am J Trop Med Hyg 1970;19:311-8.
18. McDade JE, Shepard CC, Redus MA, Newhouse VF, Smith JD. Evidence of *Rickettsia prowazekii* infection in the United States. Am J Trop Med Hyg 1980;29:277- 284.
19. Dumler JS, Taylor JP, Walker DH. Clinical and laboratory features of murine typhus in Texas, 1980 through 1987. JAMA 1991;266:1365-70.
20. Williams SG, Sacci JB Jr, Schriefer ME, Anderson EM, Fujioka K, Sorvilo FJ, et al. Typhus and typhus-like rickettsiae associated with opossums and their fleas in Los Angeles County, California. J Clin Microbiol 1992;30:1758-62.
21. Sorvillo FJ, Gondo B, Emmons R, Ryan P, Waterman SH, Tilzer A, et al. A suburban focus of endemic typhus in Los Angeles County: association with seropositive domestic cats and opossums. Am J Trop Med Hyg 1993;48:269-73.
22. Schriefer ME, Sacci JB Jr, Dumler JS, Bullen MG, Azad AF. Identification of a novel rickettsial infection in a patient diagnosed with murine typhus. J Clin Microbiol 1994;32:949-54.
23. Schriefer ME, Sacci JB Jr, Higgins JA, Taylor JP, Azad AF. Murine typhus: updated role of multiple urban components and a second typhus-like rickettsiae. J Med Entomol 1994;31:681-5.
24. Higgins JA, Radulovic S, Schriefer ME, Azad AF. *Rickettsia felis*: a new species of pathogenic rickettsia isolated from cat fleas. J Clin Microbiol 1996;34:671-4.
25. Noden BH, Radulovic S, Higgins JA, Azad AF. Molecular identification of two closely related rickettsial species, *Rickettsia typhi* and *R. felis*, in individual cat fleas, *Ctenocephalides felis* (Siphonaptera: Pulicidae). J Med Entomol. In press 1998.
26. Pancholi P, Kolbert CP, Mitchell, PD, Reed KD, Dumler JS, Bakken JS, et al. Ixodes dammini as a potential vector of human granulocytic ehrlichiosis. J Infect Dis 1995;1007-12.
27. Werren JH, Hurst GDD, Zhang W, Breeuwer JAJ, Stouthamer R, Majerus MEN. Rickettsial relative associated with male killing in the ladybird beetle (*Adalia bipunctata*). J Bacteriol 1994;176:388-94.
28. Higgins JA, Sacci JB Jr, Schriefer ME, Endris RG, Azad AF. Molecular identification of rickettsia-like microorganisms associated with colonized cat fleas (*Ctenocephalides felis*). Insect Molecular Biology 1994;3:27-33.
29. Vaughan JA, Azad AF. Acquisition of murine typhus rickettsiae by fleas. Ann N Y Acad Sci 1990;590:70-5.
30. Munderloh UG, Kurtti TJ. Cellular and molecular interrelationships between ticks and prokaryotic tick-borne pathogens. Annu Rev Entomol 1995;40:221-43.
31. Hackstadt T. The biology of rickettsiae. Infect Agents Dis 1996;5:127-43.
